# Primary Care Visit Frequency Is Associated With Diagnosis But Not Pharmacotherapy Prescribing for Patients With Alcohol Use Disorder: AUD Pharmacotherapy Prescribing in Primary Care

**DOI:** 10.1007/s11414-025-09942-6

**Published:** 2025-03-24

**Authors:** Ruth A Bishop, Ralph Ward, Andrew Schreiner, Jenna L McCauley, William P Moran, Sarah Ball

**Affiliations:** 1 Department of Internal Medicine, Medical University of South Carolina, Charleston, SC USA; 2 Department of Psychiatry and Behavioral Sciences, Medical University of South Carolina, Charleston, SC USA; 3 Department of Public Health Sciences, Medical University of South Carolina, Charleston, SC USA

**Keywords:** Alcohol use disorder, AUD, Medication for alcohol use disorder, MAUD, Medicaid

## Abstract

Primary care has been proposed as an ideal setting for the management of alcohol use disorder (AUD); however, there is limited research on the diagnosis and prescribing patterns of medications for alcohol use disorder (MAUD) within primary care. This retrospective study aims to determine whether primary care engagement is associated with the likelihood of an AUD diagnosis or prescription of MAUD. Analyzing administrative claims data from a statewide cohort of 10,138 Medicaid enrollees, only 5.9% of patients diagnosed with AUD were prescribed MAUD (including naltrexone, acamprosate, disulfiram, and topiramate). Patients with higher levels of primary care visit frequency were significantly more likely to carry an AUD diagnosis (*p* < .0001); however, primary care visit frequency was not associated with prescription of MAUD. This study highlights the underutilization of MAUD within primary care, and the need for research identifying successful strategies to address barriers to prescribing MAUD in this setting.

## Introduction

Alcohol use disorder (AUD) is the most common substance use disorder in the United States, resulting in significant morbidity and mortality. In 2022, it was estimated that 11.2% of US adults over the age of 18 met criteria for AUD.^1^ The economic costs of excessive alcohol use in the USA in 2019 were estimated to be $249 billion (about $770 per person in the USA) due to lost work productivity, motor vehicle crashes, criminal justice involvement, and healthcare expenses.^2,3^ It is estimated that over 140,000 people in the USA die annually from alcohol-related causes (annual average from 2014 to 2019), making it the fourth-leading preventable cause of death following tobacco use, unhealthy diet and insufficient exercise, and illicit substance use.^4,5^

FDA-approved pharmacotherapies for AUD include naltrexone, disulfiram, and acamprosate. Other non-FDA approved drugs including topiramate, gabapentin, and baclofen are also commonly used as medications for alcohol use disorder (MAUD).^6,7^ These therapies have been shown to be effective in reducing heavy consumption and achieving sobriety^7^ and are considered standards of care by many guidelines.^8,9^ Despite their availability and effectiveness, prescribing rates of pharmacotherapies for AUD remain low and present an area of both research and policy interest.^10–12^ Studies estimate that MAUD prescribing is quite low; only 2.1 to 9% of patients needing treatment for AUD received a prescription of one of the FDA-approved medications, which has also been found to be the case in primary care settings.^11,13–15^

Primary care has been proposed as an ideal setting for at-risk alcohol screening and prescription of MAUD given the longitudinal relationships developed between clinicians and patients and limited access to addiction specialists. Primary care may also be an ideal setting for perioperative screening of AUD given that excessive alcohol use is a risk factor for postoperative complications,^16,17^ and surgical patients are rarely offered alcohol screening or intervention referrals prior to surgery.^18,19^

Current literature suggests that physicians, including primary care physicians (PCPs), under-prescribe MAUD as it is estimated that only 1.9–5% of patients needing treatment for AUD received a prescription.^20–22^ This low prescribing rate is in spite of reimbursement for alcohol screening and treatment by Medicare and Medicaid, and several guidelines, such as the US Preventative Task Force, recommending evaluation and management of AUD.^23^ Further research is needed to provide estimates of primary care prescribing rates of these medications and barriers limiting their prescription.

This study examined the prevalence of AUD diagnoses in Medicaid claims among a South Carolina cohort. This study’s primary objectives were to identify whether patients with more frequent primary care visits were more likely to be diagnosed with AUD or receive pharmacotherapies for the treatment of AUD, while controlling for factors such as patient’s age, race/ethnicity, sex, and presence of opioid use disorder.

## Methods

This was a retrospective study involving a South Carolina Medicaid surgerical cohort originally formed as part of a parent study that examined post-surgical opioid trajectories.^28^ A surgical cohort was used to answer this question because of the robust nature of the dataset and the assumption that preoperative alcohol consumption is associated with postoperative complications, and thus, alcohol use in perioperative care would be more likely addressed in this sample than in the general population.^26,27^ Patients were included if they were insured by Medicaid and underwent a surgical procedure between January 1, 2014, and December 31, 2017. Each patient’s index time was set at 6 months prior to the earliest surgery within this period, and each was followed through the remaining study timeframe. Surgeries were identified by International Classification of Diseases (ICD)−9/10-PCS procedure codes or Healthcare Common Procedure Coding System (HCPCS) codes and included procedures for appendectomy, cholecystectomy, colorectal surgery, lumbar spine surgery, total knee replacement, and tonsillectomy. Patients were excluded if (i) they were < 13 years of age at the time of the surgical procedure, (ii) had an interruption in Medicaid eligibility during the period 6 months prior until 9 months after surgery, (iii) had no pharmacy claims during the same period, (iv) had any diagnosis code involving sickle cell disease or cancer, or (v) had a second surgery or hospitalization between 1 and 9 months after their index surgery. Data were provided by the South Carolina Revenue and Fiscal Affairs Office and included Medicaid claims and associated Medicaid pharmacy and Medicare part-D claims. The Institutional Review Board at the author’s institution (IRB study #Pro00129391) approved this study.

### Outcomes

Primary outcomes were receipt of an AUD diagnosis and prescription of MAUD during the study period. AUD diagnoses were identified from claims with a primary, secondary, or admitting diagnosis code contained in the ICD-9-CM or ICD-10-CM Elixhauser definitions for alcohol abuse as updated by Quan et al.^29^ MAUD was identified based on one or more pharmacy claims for an FDA-approved medication for AUD treatment, which included formulations of acamprosate, disulfiram, and naltrexone.^30^ Topiramate use was considered an active AUD treatment when it occurred within 90 days following an AUD diagnosis in patients without concurrent FDA-approved AUD treatment medications. Other pharmacotherapy options for AUD such as gabapentin and baclofen were not included given their frequent use for non-AUD conditions (e.g., migraine headache, neuropathic pain).

### Exposure

The primary exposure was primary care visit frequency, based on the number of visits with a primary care-designated clinician. Exposure level was a five-level categorical variable based on quartiles of annual primary care visits: none (no visits), low (0–25th percentiles; ≤ 3.9 visits/year), medium (26th–50th percentiles; 3.9–6.5 visits/year), high (51st–75th percentiles; 6.5–10.4 visits/year), and highest (76th–100th percentiles; > 10.4 visits/year). Primary care providers were identified when either the primary or secondary Medicaid provider specialty description was consistent with primary care, e.g., “family practice.” A full listing of primary care descriptions is included in the supplementary information section.

### Covariates

Demographic covariates included age (< 18, 18–31, 32–48, 49–65, and > 65 years), sex (female, male), and race/ethnicity (non-Hispanic Black, Hispanic, non-Hispanic White, Other/Unknown). Clinical variables included whether an AUD diagnosis or a MAUD claim could be linked to a primary care visit. Diagnosis linkage required the same visit claim to include a PCP and an AUD diagnosis code. MAUD linkage required a pharmacy claim within 90 days following the visit claim. Given the original intent of the dataset, this study also included categorical variables for surgery type (appendectomy, cholecystectomy, colorectal surgery, lumbar spine surgery, total knee replacement, and tonsillectomy), diagnosis of opioid use disorder (Yes, No), and chronic use of opioid pharmacotherapies prior to and after surgery (Yes, No; defined as at least one opioid prescription filled during (1) the 180 days prior and (2) ≥ 90 to 270 days following the surgical procedure).

### Statistical Analyses

Summary statistics and univariate analyses were used to describe associations between AUD diagnosis and primary care visit frequency, receipt of MAUD, and other covariates using chi-square or Fisher’s exact tests, as appropriate. Multivariable logistic models were developed for (1) the AUD diagnosis outcome using the full cohort and (2) the prescription of MAUD outcome using a population limited to patients with an AUD diagnosis. The exposure in both models was primary care visit frequency; both models were adjusted for the covariates described above. Models were checked for multicollinearity using the variance inflation factor. SAS 9.4 was used for all statistical analyses (SAS Institute, Inc., Cary, NC).

## Results

The study cohort included 10,138 unique patients of whom 30% were age 18–31 years, 78% were female, and 52% were non-Hispanic White (Table 1). Patients attended 197,801 primary care visits during the study period and were followed for a median of 2.8 years (interquartile range (IQR): 1.5, 3.6). The median number of primary care visits per year for the study cohort was 6.2 (IQR: 3.5, 10.1). In total, 512 (5.1%) patients received a diagnosis for AUD, of which 227 (44.3%) received this diagnosis during a primary care visit. Of those with a diagnosis of AUD, 30 (5.9%) had a pharmacy claim for MAUD. Six (1.2%) of these patients were prescribed naltrexone, three (0.6%) acamprosate, three (0.6%) disulfiram, and 18 (3.5%) topiramate.

**Table 1:** Characteristics of the South Carolina Medicaid surgical cohort

Variable, *N* (%)	Characteristic	AUD	No AUD	Total	*p*-value
Group size		512 (5.1%)	9626 (95%)	10,138	
AUD medications prescribed?*	Naltrexone	6 (1.2%)	14 (0.1%)	20 (0.2%)	< .0001
	Acamprosate	3 (0.6%)	0 (0%)	3 (0.03%)	
	Disulfiram	3 (0.6%)	0 (0%)	3 (0.03%)	
	Topiramate	18 (3.5%)	0 (0%)	18 (0.2%)	
AUD diagnosis linked to PCP visit?	Yes	227 (44.3%)	0 (0%)	227 (2.2%)	< .0001
AUD medications linked to AUD Dx visit?	Yes	25 (4.9%)	0 (0%)	25 (0.2%)	< .0001
Primary care visit frequency**	None	9 (1.8%)	494 (5.1%)	503 (5.0%)	< .0001
	Low	89 (17.4%)	2388 (24.8%)	2477 (24.4%)	
	Medium	131 (25.6%)	2213 (23%)	2344 (23.1%)	
	High	128 (25%)	2303 (23.9%)	2431 (24.0%)	
	Highest	155 (30.3%)	2228 (23.1%)	2383 (23.5%)	
Sex	Female	310 (60.5%)	7616 (79.1%)	7926 (78.2%)	< .0001
	Male	202 (39.5%)	2010 (20.9%)	2212 (21.8%)	
Age group (in years)	< 18	29 (5.7%)	1909 (19.8%)	1938 (19.1%)	< .0001
	18–31	131 (25.6%)	2908 (30.2%)	3039 (30.0%)	
	32–48	142 (27.7%)	2402 (25%)	2544 (25.1%)	
	49–64	192 (37.5%)	1748 (18.2%)	1940 (19.1%)	
	> = 65	18 (3.5%)	659 (6.8%)	677 (6.7%)	
Race/ethnicity	Black	144 (28.1%)	3299 (34.3%)	3443 (40.0%)	< .0001
	Hispanic	6 (1.2%)	315 (3.3%)	321 (3.2%)	
	Other/Unknown	88 (17.2%)	1047 (10.9%)	1135 (11.2%)	
	White	274 (53.5%)	4965 (51.6%)	5239 (51.7%)	
Chronic opioid use pre- and post-surgery	Yes	228 (44.5%)	2731 (28.4%)	2959 (29.2%)	< .0001
OUD diagnosis	Yes	34 (6.6%)	163 (1.7%)	197 (1.9%)	< .0001
Surgery type	Appendectomy	64 (12.5%)	1335 (13.9%)	1399 (13.8%)	< .0001
	Cholecystectomy	254 (49.6%)	4661 (48.4%)	4915 (48.5%)	
	Colon	22 (4.3%)	154 (1.6%)	176 (1.7%)	
	Lumbar spine	72 (14.1%)	1060 (11%)	1132 (11.2%)	
	Total knee replacement	78 (15.2%)	1208 (12.5%)	1286 (12.7%)	
	Tonsillectomy	22 (4.3%)	1208 (12.5%)	1230 (12.1%)	

*Naltrexone, acamprosate, or disulfiram at any point, or topiramate within 90 days following AUD diagnosis^**^Primary care visit frequency: low ≤ 3.9 visits/year; medium > 3.9 and ≤ 6.5 visit/year; high > 6.5 and ≤ 10.4 visits/year; highest > 10.4 visits/year

Patients with higher levels of primary care visit frequency were significantly more likely to be diagnosed with AUD (*p* < 0.0001). Men were more likely to receive an AUD diagnosis compared to women (9.3% vs. 3.9%, *p* < 0.0001). Those with an AUD diagnosis were more likely to be chronic opioid users before and after surgery (44.5% vs. 28.4% for non-AUD patients, *p* < 0.0001) and were more likely to carry an opioid use disorder diagnosis (6.6% vs. 1.7%, *p* < 0.0001). Patient age, race and ethnicity, and surgery also differed by AUD diagnosis group.

In the multivariable logistic regression model for receipt of an AUD diagnosis, patients in the medium (> 3.9 and ≤ 6.5 visits/year), high (> 6.5 and ≤ 10.4 visits/year), and highest (> 10.4 visits/year) quartiles of primary care visit frequency all had more than twice the odds for having an AUD diagnosis compared to those with no primary care visits: (adjusted odds ratio (AOR): 2.55, 95% confidence interval (CI): 1.26, 5.17; *p* = 0.009 for highest frequency level) (Table 2). Female patients had 65% lower odds for an AUD diagnosis compared to men (AOR 0.35; 95% CI 0.29–0.43; *p* < 0.0001); however, they were significantly more likely to have pharmacy claims for MAUD if they carried an AUD diagnosis (AOR: 2.86; 95% CI 1.07, 7.67; *p* < 0.0001). An OUD diagnosis was associated with substantially greater odds of an AUD diagnosis (AOR: 3.03; 95% CI 2.03, 4.51; *p* < 0.0001). Primary care frequency was not associated with receipt of MAUD among patients with AUD.

**Table 2:** Logistic regression models for AUD diagnosis (Model 1) and fulfillment of a prescription for AUD pharmacotherapy among patients with an AUD diagnosis (Model 2)

Variable	Characteristic	Model 1		Model 2	
		AUD diagnosis, *n* = 10,138		MAUD prescription, *n* = 512	
		*p*-value	AOR (95% CI)	*p*-value	AOR (95% CI)
Primary care visit frequency*** ref = none	Low	0.14	1.71 (0.84, 3.46)	0.79	0.71 (0.06, 8.57)
	Medium	0.02	2.31 (1.14, 4.69)	0.42	0.35 (0.03, 4.25)
	High	0.038	2.12 (1.04, 4.30)	0.52	0.43 (0.03, 5.39)
	Highest	0.009	2.55 (1.26, 5.17)	0.45	0.38 (0.03, 4.66)
Race and ethnicity ref = “White”	Non-Hispanic White	ref	ref	ref	ref
	Black	0.38	0.91 (0.73, 1.13)	0.13	0.52 (0.22, 1.20)
	Hispanic	0.12	0.52 (0.23, 1.18)	*	
	Other/Unknown	0.054	1.29 (1.00, 1.68)		
Age group ref ≤ 18 years	18 to 31 years	< .0001	2.87 (1.84, 4.47)	0.65	0.64 (0.09, 4.51)
	32 to 48 years	< .0001	3.18 (2.02, 4.98)	0.08	4.52 (0.86, 23.9)
	49 to 64 years	< .0001	5.01 (3.15, 7.97)	0.8	1.26 (0.21, 7.55)
	65 years or older	0.16	1.59 (0.83, 3.03)	**	
Sex (ref = male)	Female	< .0001	0.35 (0.29, 0.43)	0.037	2.86 (1.07, 7.67)
Surgery type ref = tonsillectomy	Appendectomy	0.029	1.75 (1.06, 2.89)	0.36	0.40 (0.05, 2.87)
	Cholecystectomy	0.051	1.60 (1.00, 2.55)	0.46	0.52 (0.09, 2.93)
	Colon	0.005	2.55 (1.32, 4.91)	0.68	0.57 (0.04, 8.33)
	Lumbar spine	0.61	1.15 (0.68, 1.95)	0.88	1.15 (0.17, 7.83)
	Total knee replacement	0.55	1.18 (0.69, 2.03)	0.64	0.61 (0.08, 4.80)
Chronic opioid use pre- and post-surgery	Yes vs No	0.003	1.36 (1.11, 1.68)	0.47	0.72 (0.29, 1.78)
Opioid use disorder	Yes vs No	< .0001	3.03 (2.03, 4.51)	0.68	0.72 (0.15, 3.42)

*CI* confidence interval, *AOR* adjusted odds ratio*In model 2: Black, Hispanic, and Other groups combined to form minority groups due to smaller AUD population**In model 2: 65 and older group combined with 49 to 64 group***Primary care visit frequency: low ≤ 3.9 visits/year; medium > 3.9 and ≤ 6.5 visits/year; high > 6.5 and ≤ 10.4 visits/year; highest > 10.4 visits/year

## Discussion

In this study examining the relationship between primary care engagement and AUD diagnosis/MAUD prescribing, primary care engagement was associated with a higher probability of receiving an AUD diagnosis, but not pharmacotherapy, for AUD. The estimated prevalence of AUD in this cohort was 5.1%, slightly lower than the national prevalence of AUD estimated by SAMHSA’s National Survey on Drug Use and Health (NSDUH) during the study period 2014–2017, which ranged from 5.3 to 6.4%. Of those diagnosed with AUD, only 5.9% had an associated pharmacy claim for MAUD, suggesting that most patients with AUD do not receive guideline-based treatment.^8,20,31,32^

Low rates of MAUD prescribing in this study are similar to other national and Veterans Health Administration studies both within and outside of primary care, suggesting broad underutilization of these medications in clinical practice at large.^11,13–15,20,31–33^ This study replicates this finding within a Medicaid surgical cohort well-connected to primary care (median 6.2 primary care visits per year).

While there is relatively limited literature describing the relationship between primary care visits and AUD diagnosis, several pre-existing studies suggest a similar relationship in that overall, AUD screening in primary care is low, but increased engagement with primary care is associated with an increased likelihood of AUD diagnosis.^15,34^ A cross-sectional analysis of the National Ambulatory Medical Care Survey data collected from 2014 to 2016 demonstrated that alcohol screening occurred during 2.6% of visits and counseling was documented in just 0.8% of visits. Despite these low rates of screening and treatment intervention, this study showed that screening was more likely if the patient was seen by their assigned primary care physician.^34^

Studies examining the relationship between primary care engagement and MAUD prescribing are also limited and demonstrate mixed results. One retrospective cohort study among primary care patients from a large, urban academic medical center demonstrated that patients engaged with primary care had a slightly higher probability of being prescribed MAUD compared to patients with limited primary care engagement (2.89% versus 2.56%).^15^ However, other studies have shown negative correlations between MAUD prescribing and primary care as well as large variations in individual primary care physician’s prescription rates, suggesting that a small number of providers are responsible for the majority of MAUD prescriptions.^20,21^

Barriers to MAUD prescribing at the primary care provider level described in the literature include providers’ lack of education on MAUD, provider’s negative beliefs about medication effectiveness or perceived poor patient adherence, and competing demands within primary care that make prescribing MAUD a lower priority.^21,35–37^ This study highlights the need for evidence-based strategies to improve MAUD prescribing in primary care.

This study has several limitations. This is a surgical, Medicaid cohort study; as Medicaid eligibility varies by state, study findings may not be generalizable to other states’ Medicaid populations, to non-Medicaid populations, or to non-surgical populations. Additionally, it is possible that current AUD diagnosis and MAUD prescribing practices differ from 2014 to 2017, when this data was collected. Another limitation is this study examined prescriptions for MAUD but did not capture data on ambulatory screening or other types of treatment such as behavioral interventions. This study also did not capture treatment outcomes, adherence, or duration of treatment and excluded other medications that may have been prescribed for AUD including baclofen or gabapentin. Lastly, it is possible that not all patients with AUD or prescriptions for MAUD were captured within the cohort given that ICD-9/10 codes can have limited sensitivity for detecting clinical conditions in administrative data.^38^

## Implications for Behavioral Health

The findings of this study indicate that a significant number of patients with AUD do not receive evidence-based pharmacotherapy treatments. Patients who saw their primary care provider more frequently were more likely to be diagnosed with AUD, suggesting that PCPs routinely identify AUD. However, the study revealed no positive correlation between the frequency of primary care visits and the prescription of medications for alcohol use disorder (MAUD), indicating that PCPs often fail to offer pharmacological treatment even when AUD is diagnosed.

The underutilization of MAUD in primary care specialties^39^ underscores the need for future research to explore the barriers PCPs face when prescribing MAUD as well as implementation strategies to overcome these challenges. Future research should also aim to identify characteristics of patients that would make them the most appropriate candidates for MAUD. Given the increase in prevalence of AUD seen in the post-COVID era,^1^ these studies are needed now more than ever, offering a promising opportunity to improve outcomes for this common, costly, and devastating disease.

## Data Availability

The data that support the findings of this study are not openly available due to reasons of sensitivity but are available from the corresponding author upon reasonable request. Data are located in controlled access data storage at the Medical University of South Carolina.

## Declarations

### Disclaimer

SCDHHS had no role in the preparation, approval, or decision to submit the manuscript for publication.
